# The effect of ambient ozone exposure on three types of diabetes: a meta-analysis

**DOI:** 10.1186/s12940-023-00981-0

**Published:** 2023-03-30

**Authors:** Sirui Yu, Mingzhi Zhang, Jiamin Zhu, Xu Yang, Francis Manyori Bigambo, Antoine M. Snijders, Xu Wang, Weiyue Hu, Wei Lv, Yankai Xia

**Affiliations:** 1 State Key Laboratory of Reproductive Medicine, Center for Global Health, Institute of Toxicology, School of Public Health, Nanjing Medical University, No.101 Longmian Road, Nanjing, 211166 China; 2 Key Laboratory of Modern Toxicology of Ministry of Education, School of Public Health, Nanjing Medical University, Nanjing, Jiangsu China; 3 Biological Systems and Engineering Division, Lawrence Berkeley National Laboratory, Berkeley, CA 94720 USA; 4 Department of Endocrinology, Children’s Hospital of Nanjing Medical University, Nanjing, Jiangsu China; 5 Department of Nutrition and Food Safety, School of Public Health, Nanjing Medical University, No.101 Longmian Road, Nanjing, 211166 China; 6 Healthcare Management Program, School of Business, Nanjing University, 22 Hankou Rd, Nanjing, 210093 China

**Keywords:** O_3_, Air pollution, T1D, T2D, GDM, Systematic review

## Abstract

**Background:**

Ozone as an air pollutant is gradually becoming a threat to people's health. However, the effect of ozone exposure on risk of developing diabetes, a fast-growing global metabolic disease, remains controversial.

**Objective:**

To evaluate the impact of ambient ozone exposure on the incidence rate of type 1, type 2 and gestational diabetes mellitus.

**Method:**

We systematically searched PubMed, Web of Science, and Cochrane Library databases before July 9, 2022, to determine relevant literature. Data were extracted after quality evaluation according to the Newcastle Ottawa Scale (NOS) and the agency for healthcare research and quality (AHRQ) standards, and a meta-analysis was used to evaluate the correlation between ozone exposure and type 1 diabetes mellitus (T1D), type 2 diabetes mellitus (T2D), and gestational diabetes mellitus (GDM). The heterogeneity test, sensitivity analysis, and publication bias were performed using Stata 16.0.

**Results:**

Our search identified 667 studies from three databases, 19 of which were included in our analysis after removing duplicate and ineligible studies. Among the remaining studies, three were on T1D, five were on T2D, and eleven were on GDM. The result showed that ozone exposure was positively correlated with T2D [effect size (ES) = 1.06, 95% CI: 1.02, 1.11] and GDM [pooled odds ratio (OR) = 1.01, 95% CI: 1.00, 1.03]. Subgroup analysis demonstrated that ozone exposure in the first trimester of pregnancy might raise the risk of GDM. However, no significant association was observed between ozone exposure and T1D.

**Conclusion:**

Long-term exposure to ozone may increase the risk of T2D, and daily ozone exposure during pregnancy was a hazard factor for developing GDM. Decreasing ambient ozone pollution may reduce the burden of both diseases.

**Supplementary Information:**

The online version contains supplementary material available at 10.1186/s12940-023-00981-0.

## Introduction

Ozone in the troposphere is created in the presence of solar radiation, due to the reaction of nitrogen oxides and volatile organic compounds. Growing evidence have shown that high concentration of ozone exposure could threaten people’s health and might be linked to lower life expectancy [22, 32, 63]. Ozone as a typical air pollutant can exacerbate lung injury, increase the risk of respiratory diseases [39, 66], cardiovascular disease, reproductive abnormalities, as well as neurological abnormalities [50]. It is worth noting that through neuro-endocrine regulation, ozone exposure may cause metabolic syndrome, characterized by glucose intolerance and hyperlipidemia [49, 50]. Importantly, glucose intolerance often indicates pre-existing diabetes or predisposition to diabetes.

Diabetes is the most common chronic metabolic disease and its high incidence has caused a heavy medical and economic burden on society. Diabetes can be divided into the following categories: type 1 diabetes or T1D (insufficient insulin secretion), type 2 diabetes or T2D (insulin resistance with progressive insulin secretory defect), and gestational diabetes or GDM (various levels of impaired glucose tolerance which first occur or are first detected during pregnancy) [1]. T1D mainly occurs in children and adolescents. T1D is often first diagnosed from a routine blood test indicating modest hyperglycemia which then evolves into severe hyperglycemia or ketoacidosis if left untreated [12, 14]. T2D can cause devastating macrovascular complications and microvascular complications, which can cause severe sequelae, such as diabetic retinopathy, blindness, kidney failure, and neuropathy [9]. GDM as a type of metabolic disturbance during pregnancy, may cause various health risks in the mother and the child. In women, it can cause serious perinatal complications such as cardiovascular diseases and it can evolve into T2D after pregnancy. In women with GDM, the fetus has an increased risk of developing macrosomia, birth injury and cardiometabolic disease later in life [5, 24, 54, 65].

Chuang et al. demonstrated that increased ozone exposure was associated with increased fasting blood glucose and HbA1c levels, a biomarker of glucose metabolism [8]. Experimental evidence also indicated that ozone may cause damage to β cells [38], and exert insulin resistance, possibly due to oxidative stress and inflammatory responses [25]. These suggest that ozone exposure may lead to the appropriate type of diabetes in different populations.

However, the epidemiological evidence of ozone exposure on three types of diabetes still remains controversial. Evidence shows that ozone exposure increases the overall prevalence of diabetes [40], however, existing epidemiological studies suggest that increased ozone exposure is associated with a decrease in diabetes prevalence, which persists after adjusting for possible confounding factors [29, 52]. Hathout et al. found the positive correlation between ozone and T1D [18], but a negative correlation was observed by Elten et al. [11]. A study in areas with low average ozone exposure found significant positive effects [21], but not in areas with higher levels [62]. Results of studies also varies on the association between GDM and ozone exposure [19, 41]. Overall, findings on the association between ozone exposure and the three types of diabetes are inconsistent, which may depend on study design, sample size, exposure measurement methods, and outcome assessment.

At present, direct evidence on the relationship between different types of diabetes and ozone exposure is still being studied. Integration of the results of current studies on this topic is urgently needed to obtain more representative and reliable conclusions with a larger sample size and a wider study area, and stronger statistical power. Thus, a meta-analysis was conducted to explore whether ozone exposure is associated with three types of diabetes.

In this study, we performed a meta-analysis to evaluate the relationship of ozone exposure to T1D, T2D, and GDM, aimed to provide evidence for the potentially harmful effects of ozone. In addition, impacts on average ozone concentration, socioeconomic status, exposure measurement methods on T2D and GDM were also investigated via subgroup analyses.

## Material and methods

### Search methods

This study was conducted according to the Preferred Reporting Items for Systematic Reviews and Meta-Analysis (PRISMA) guidelines (Supplemental Table S2). Relevant articles were retrieved from three databases: *Web of Science*, *PubMed* and *Cochrane Library* up to July 9, 2022. According to the PECO (Patients, Intervention, Comparison, Outcomes), we have defined eligible studies as follows. P: People with three types of diabetes; E: Ozone exposure before illness; C: People who did not have diabetes and have a negative glucose tolerance test; O: The documented result is the development of the specific type of diabetes. The following search terms were used to screen the articles across three databases, and three types of diabetes were retrieved separately:

- #1: (ozone) OR (O_3_)
- #2: (Type 1 Diabetes) OR (Insulin-Dependent Diabetes Mellitus) OR (Diabetes Mellitus, Type 1) OR T1D
- #3: (Type 2 Diabetes) OR (Non-Insulin-Dependent Diabetes Mellitus) OR (Diabetes Mellitus, Type 2) OR T2D
- #4: (Pregnancy-Induced Diabetes) OR (Gestational Diabetes Mellitus) OR (GDM)
- #5: #1 AND #2 (#1 AND #3 or #1 AND #4)

We also manually searched the list of references to ensure that there were no omissions.

### Selection criteria

The criteria of inclusion and exclusion were as follows:

Inclusion criteria:

1. Epidemiological studies were based on observation and analysis such as cohort studies, case–control studies, and cross-sectional studies;
2. Exposure factor was ozone;
3. The outcome was the correlation between ozone exposure and the risk of diabetes mellitus;
4. Data, such as OR, risk ratio (RR), hazard ratio (HR), and 95% CI (confidence interval), were provided in the study.

Exclusion Criteria:

1. Animal studies, reviews, conference abstracts, systematic reviews, and meta-analyses;
2. Studies that did not fit into the research topic;
3. Incomplete articles, including lack of statistical analysis details;
4. Studies with low quality score < 7. For example, there is a lack of key covariates or studies in which covariates differ significantly from other studies.

### Study screening and data extraction

Articles were imported into Endnote for management, and duplicates were removed. We manually screened the retrieved articles by the title and abstract based on the inclusion and exclusion criteria. In addition, full texts were reviewed for further confirmation and the acquisition of data. Two researchers independently completed the literature screening process. The data extracted included: the first author's name, published year, country, study design, sample size, participants’ age, ozone exposure period, type of diabetes, effect size, and 95% CI.

### Quality assessment

NOS was used to evaluate the quality of the cohort and case–control studies included in this analysis, a score of 7 was considered a high-quality article. In addition, the 11-items standard recommended by the AHRQ was used to evaluate the cross-sectional studies [64]. The literature was divided into levels as follows: A score of 0–3 was considered low quality, a score of 4–7 was moderate quality, and a score of 8–11 was high quality [20].We used the Grading of Recommendations Assessment, Development and Evaluation (GRADE) approach to assess the evidence level for each outcome [4].

### Statistical methods

STATA version 16.0 was used to perform statistical analyses. Inclusive HRs, ORs and their 95% CI were fed into the package for effect merging. All effect values were included in the same meta-analysis and the ES was used to estimate the overall effect [2].

During analyses, there were specific steps: (1) Standardization: Inclusive effect values were normalized to 10 μg/m^3^ as the unit of increase. (2) Unit conversation: Considering some articles used ppb as unit, we refer to WHO for the method to convert research into the same indicators, that is, the conversion coefficient from parts per billion (ppb) to μg/m^3^ (1 ppb = 1.96 μg/m^3^ ozone) [26]. The following formula was applied to recalculate the RR for the standardized increment [27]:

$${RR}_{Standardized}=e^{\left(\frac{\ln\left({RR}_{Origin}\right)}{{Increment}_{Origin}}\times\;{Increment}_{Standardized}\right)}$$

(3) Heterogeneity test was measured by I^2^ statistic: If I^2^ > 50% or *p* < 0.05, the value of combined effects was calculated using the random effects model (REM) to reduce the significant heterogeneity, which was visualized with a forest plot. The REM estimates confidence intervals based on sampling error within studies and variation between studies. When heterogeneity was statistically significant, REM was more conservative and robust than the fixed-effect model. The DerSimonian-Laird method was used, which encompasses the variability within and between studies [47]. (4) Subgroup analysis: In articles of gestational diabetes, subgroup analysis was performed based on trimesters exposure to ozone to reduce heterogeneity. The impacts of average ozone concentration, socioeconomic status, exposure measurement methods on T2D and GDM were also investigated via subgroup analyses. (5) Test and correction of publication bias: Publication bias was tested by Begg's Test and Egger's Test, and was visualized using funnel plots. (6) Sensitivity analysis: In order to assess the reliability of studies included in this meta-analysis, each article was excluded one by one for sensitivity analysis.

## Results

### Study search results

**Fig. 1:**
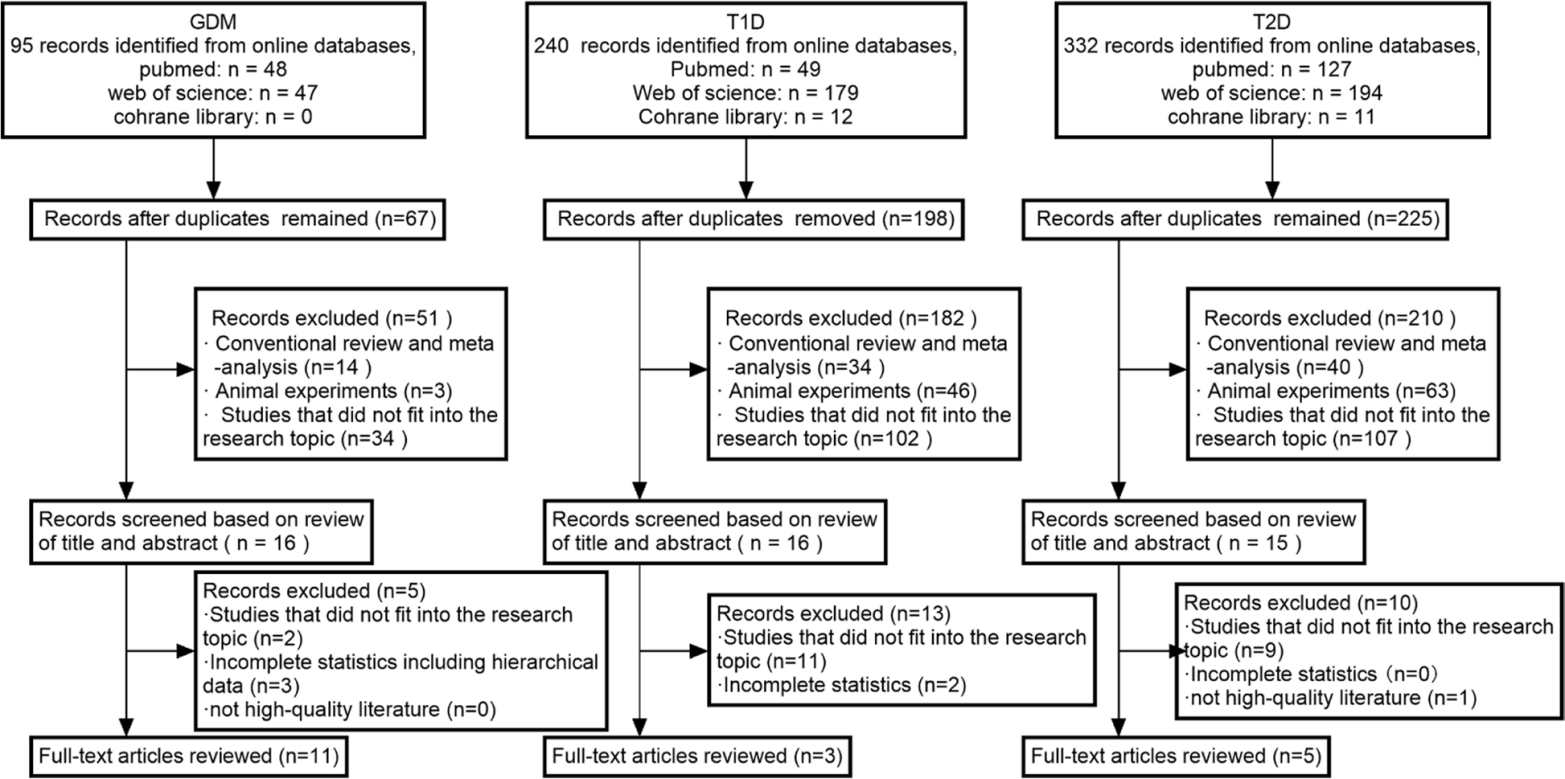
The flowchart of study screening and selection

The study screening process is shown in Fig. 1. Three types of diabetes were separately retrieved based on the search strategy, and a total of 667 records were initially identified (T1D 240, T2D 332, GDM 95).

#### T1D

A total of 16 records entered the next round of screening after duplicate verification and summary assessment. Through further review of the full-text and removing articles of irrelevant exposure or outcome, three studies were included in our analysis [11, 17, 18]. Since there was a deficiency of articles on exposure during pregnancy, we adopted three articles on childhood exposure (Table 1, Supplemental Table S1).

**Table 1:** Characteristics of included literature and quality evaluation

Author	Year	Country/region	Study design	Sample size	Age at diagnosis (years)	Exposure period	Covariate	Outcome	Quality assessment score	PMID
Elten et al	2020	Canada	Cohort study	754,698	< 6	trimesters, childhood	pollutant, sex, maternal age at delivery, smoking,birth weight parity, gestational age	T1D	7	32,120,123
Hathout et al	2002	America	Case–control study	110	< 18	childhood	age		7	15,016,145
Hathout et al	2006	America	Case–control study	402	7.4 ± 4	childhood	age		7	16,629,713
Jerrett et al	2017	America	Cohort study	453,221	≥ 30	daily 8-h maximum O_3_	smoking status, exercise, diet, parental history of diabetes, BMI, neighborhood socio-economic status (SES), education	T2D	8	28,153,529
Li et al	2021	Taiwan	Cohort study	6,426,802	65.17 ± 12.82	daily average concentrations of O_3_	age, sex, SES, urbanization level, temperature, humidity and baseline chronic comorbidity status		8	33,412,098
Renzi et al	2018	Italy	Cohort study	1,425,580	≥ 35	daily 8-h maximum O_3_	sex, SES, place of birth, occupation, educationa, preexisting comorbidities, marital status		8	29,253,730
Yang et al	2018	China	Cross-sectional study	15,477	18–74	long-term	age, sex, BMI, education, family income, smoking, alcohol consumption, diet, exercise, family history of diabetes, and district		7	29,615,239
Yu et al	2021	America	Cohort study	1,090	70.5 ± 6.9	daily 8-h maximum O_3_	age, sex, education, occupation, physical activity, smoking status, and household income at baseline		7	34,494,856
Hu et al	2015	America	Case–control study	410,267	/	trimester 1, trimester 2, entire pregnancy	maternal age, race, education, marital status, season of conception and year of delivery, median household income, prenatal care began, urbanization	GDM	8	25,794,412
Jo et al	2019	America	Cohort study	239,574	32.4 ± 5.4	preconception, trimester 1, trimester 2	maternal age, education, race, household income		8	31,234,004
Lin et al	2020	China	Cohort study	12,842	/	trimester 1, trimester 2, two trimesters	maternal age, race, education, marital status, conception season, occupation, temperature, humidity, pre-pregnancy BMI		8	32,739,627
Liu et al	2022	China	Cohort study	20,113	30	preconception, trimester 1, trimester 2	maternal age, pre-pregnancy BMI, education, family history of diabetes, parity, season of LMP, temperature		9	34,798,119
Pan et al	2017	Taiwan	Cohort study	19,606	31.9 ± 4.5	trimester 1, trimester 2, trimester 3	maternal age, BMI, weight gain, fetal gender, parity and annual household income		7	28,672,129
Robledo et al	2015	America	Cohort study	219,952	/	preconception, trimester 1	maternal age, race and study site		8	25,601,734
Shen et al	2017	Taiwan	Case–control study	13,434	31.30 ± 4.54	preconception, trimester 1, trimester 2	season of delivery, number of births, obesity, history of polycystic ovary syndrome (PCOS), personal monthly income, disease burden, median family income, level of urbanization		9	29,261,145
Sun et al	2022	America	Cohort study	395,927	30.3 ± 5.7	preconception, trimester 1, trimester 2, entire pregnancy	maternal age, race, education, family household income, pre-pregnancy BMI, smoking, insurance type, season of conception and year of delivery		8	34,563,749
Wu et al	2016	America	Cohort study	44,949	/	trimester 1, trimester 2, trimester 3	maternal age, race, education, median household income		8	29,659,239
Yao et al	2020	China	Cohort study	5,427	/	preconception, trimester 1	maternal age, education, season of blood collection, fruit and dessert intake frequency, pre-pregnancy BMI, parity, physical activity during pregnancy, family history of diabetes, temperature, and relative humidity		7	32,278,159
Yan et al	2022	China	Cohort study	3,754	29.6 ± 4.3	trimester 1, trimester 2, trimester 3, entire pregnancy	maternal age, diabetes mellitus, pre- pregnancy BMI, pre-pregnancy hypertension and residential region, sex, season of conception		8	3,567,971

“/” represent all age groups

#### T2D

After the removal of the articles with irrelevant exposure or outcome and literature quality scoring, five studies were included in our analysis [21, 34, 43, 59, 62].

#### GDM

A total of 79 records that did not meet the inclusion criteria were removed after duplicate verification and summary assessment. After further review of the remaining 16 full-text articles, five studies were removed because two studies had irrelevant exposure or outcome, another two studies had incomplete statistics, and one study had hierarchical data and could not be included in the analysis. The remaining 11 studies were included in our analysis [19, 23, 35, 36, 41, 44, 48, 51, 56, 58, 60].

### Characteristics overview

Table 1 shows the characteristics of 19 studies included in our analysis. In terms of the number of studies (N), America had 9 studies, followed by China (*N* = 5), Taiwan (*N* = 3), Italy (*N* = 1), and Canada (*N* = 1). In terms of the number of samples (n) and proportion of samples, most of the included samples were from Taiwan (*N* = 6,459,842; 62%), followed by America (*N* = 1,765,492; 17%), Italy (*N* = 1,425,580; 14%), Canada (*N* = 754,698; 7%), and China (*N* = 57,613; 0.55%). Among them, 14 studies were cohort studies, 4 were case-control studies, and 1 was a cross-sectional study. The quality of the 19 selected studies ranged from 7 to 9 according to the NOS standard, indicating that all studies were of moderate to high-quality. The initial certainty of evidence for observational studies was low. Based on study limitations, inconsistency, imprecision, indirectness and publication bias, we further adjusted the evidence certainty of these studies and presented them in Table S1. The downgrading was mainly due to imprecision. Of these, 16 of the outcomes were low and 12 of the outcomes were very low.

For the T1D studies, the sample size ranged from 110 to 754,698. Ozone exposure time was childhood, including 0–18 years of age. For the T2D studies, the sample size ranged from 1,090 to 6,426,802. Ozone exposure time was long-term and the age ranged from 18 to 75 years of age. For the GDM studies, the sample size ranged from 3,754 to 410,267. The majority of the study population was between 20–35 years of age. The diagnosis of GDM was validated through an oral glucose tolerance test (OGTT) and uniform criteria. The exposure time included preconception and the three trimesters throughout pregnancy (the 1^st^ trimester: 1–13 gestational weeks; the 2^nd^ trimester: 14–27 or 14–26 gestational weeks; and the 3^rd^ trimester: over 27 weeks of pregnancy). To reduce significant heterogeneity, subgroup analysis was performed based on the exposure time. The average value of daily 8-h maximum ozone concentration or the daily average ozone concentration was used for exposure assessment. The OR and 95% CI of eligible studies were collected after adjustment for potential confounding factors including children/gestational age, BMI, smoking, education level and race. Hathout et al. used age as an adjustment factor [17, 18]. Emphasis was placed on factors such as socio-economic status, marital status, place of birth and sex in the article of Renzi et al. [43]. We investigated the relationship between ozone exposure during childhood and T1D, daily ozone exposure and T2D, and ozone exposure during pregnancy and GDM.

### Meta- analysis on ozone exposure and the risk of diabetes

#### The association between ozone exposure and T1D

**Fig. 2:**
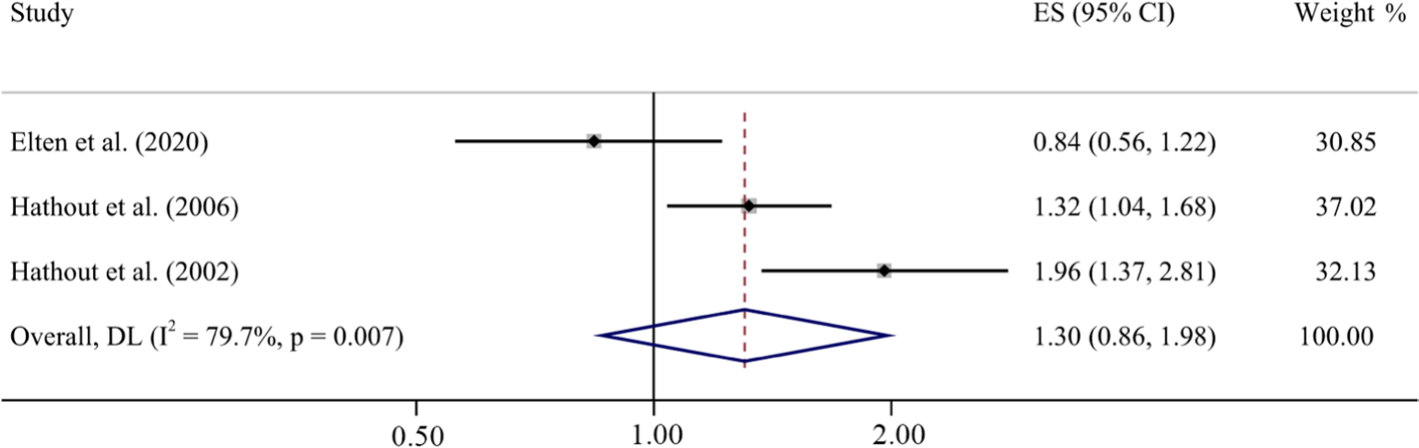
Forest plot for T1D and ozone exposure during childhood (per 10 μg/m^3^ increase)

The effectors of three studies were pooled to analyze the association between ozone exposure and T1D. The random effect model was adopted due to significant heterogeneity among these effects (tau-squared = 0.11, I^2^ = 79.7%, *p* = 0.007) (Fig. 2). The results from the forest plot showed that the increase (10 μg/m^3^) in ozone exposure in childhood was correlated with T1D, but not statistically significant (ES = 1.30, 95% CI: 0.86, 1.98).

#### The association between ozone exposure and T2D

**Fig. 3:**
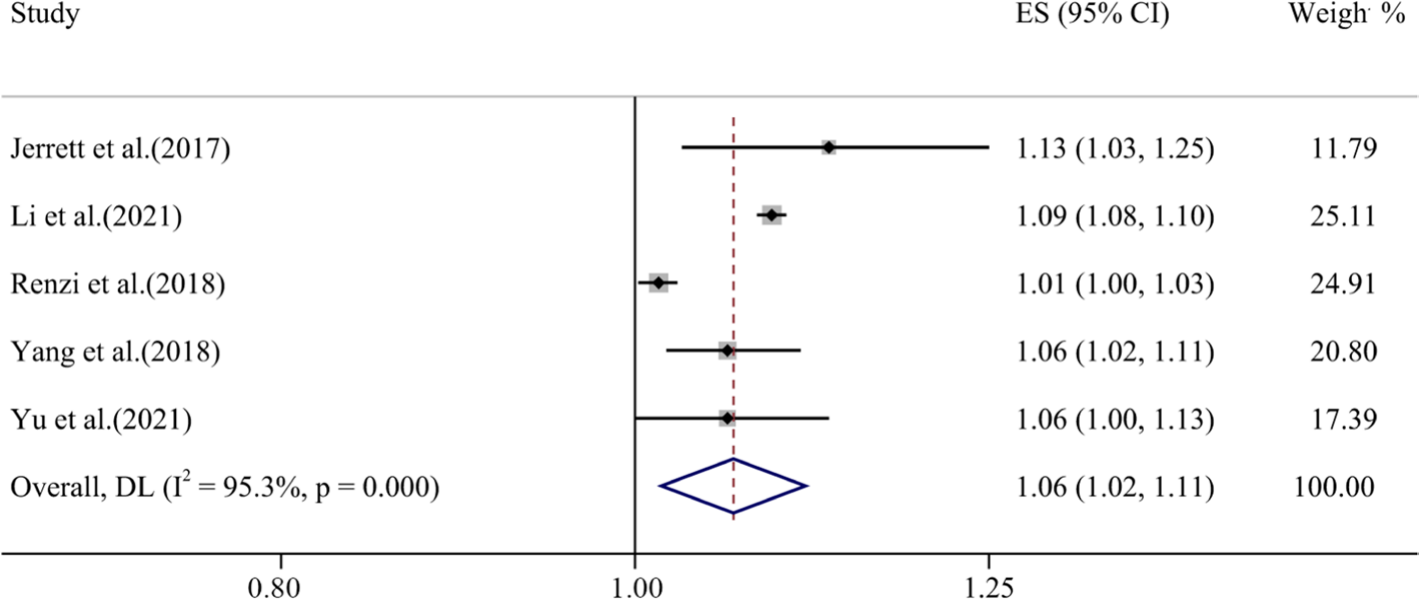
Forest plot for T2D and long-time ozone exposure (per 10 μg/m^3^ increase)

The effect size of five studies was included in the analysis and REM was utilized to represent the relationship between ozone exposure and T2D with tau-squared = 0.00, I^2^ = 95.3% (*p* < 0.001). The forest plot results showed a positive association between the increase (10 μg/m^3^) in ozone exposure and T2D, which was statistically significant (ES = 1.06, 95% CI: 1.02, 1.11) (Fig. 3).

#### The association between ozone exposure and GDM

**Fig. 4:**
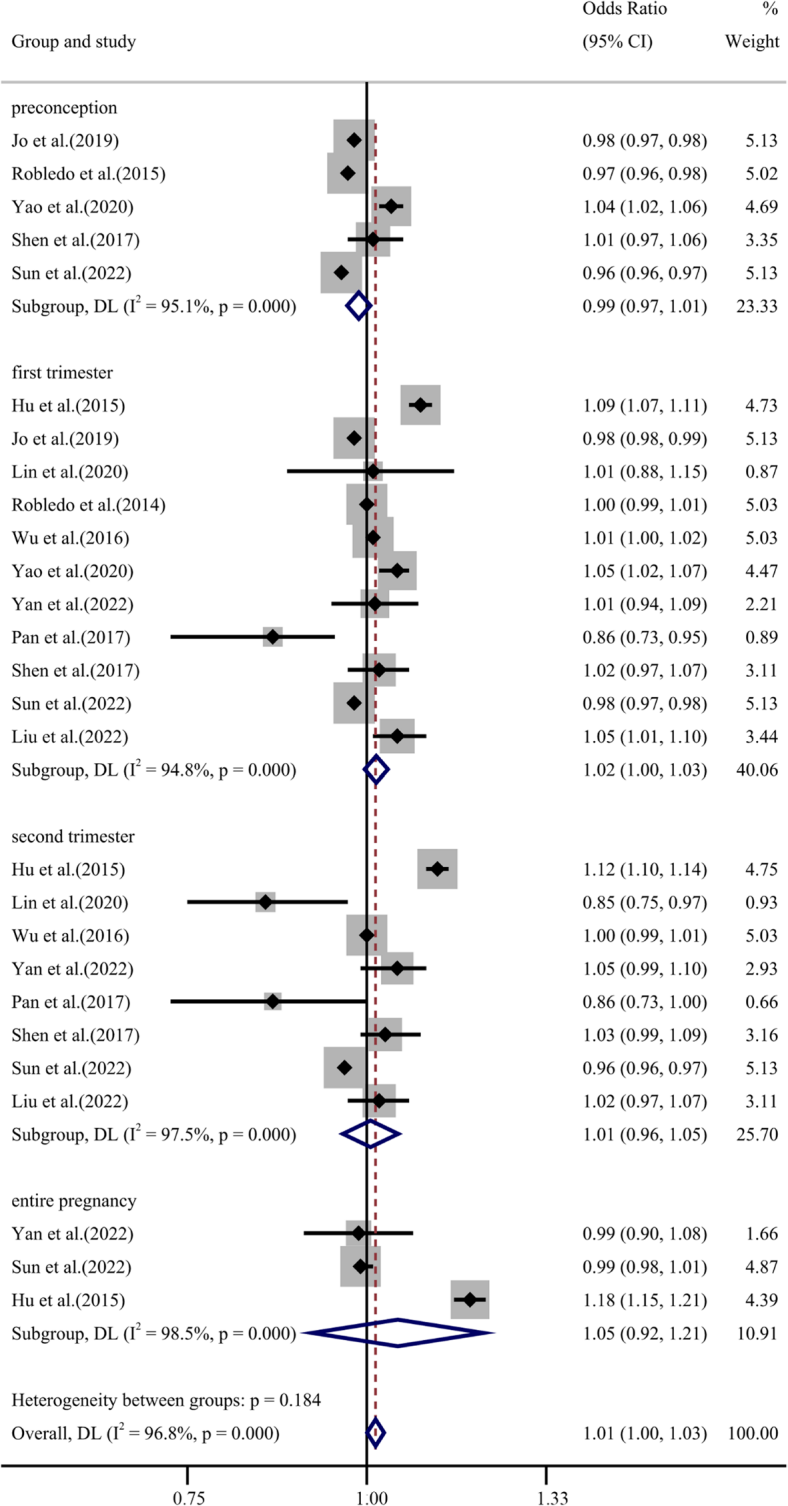
Forest plot for GDM and ozone exposure (per 10 μg/m^3^ increase) during preconception, the first trimester, the second trimester and entire pregnancy

Eleven studies were included to explore the association between ozone exposure and GDM. We performed four subgroup analyses according to exposure time. The overall results of subgroups showed that ozone exposure (per 10 μg/m^3^ increase) was associated with GDM, with the overall pooled OR = 1.01 (95% CI: 1.00, 1.03). Eleven studies were included to evaluate the association of ozone exposure in the first trimester with GDM, and the result was statistically significant (OR = 1.02, 95% CI: 1.00, 1.03) (Fig. 4). The effect sizes of the five articles, which explored the association between the second trimester ozone exposure and GDM showed no significant association with OR = 1.01 (95% CI: 0.96, 1.05). Moreover, three articles that explore the association between ozone exposure and GDM in the entire pregnancy were statistically insignificant with OR = 1.05 (95% CI: 0.92, 1.21). In addition, the effect sizes of the eight articles, which were preconception ozone exposure revealed marginal significance with OR = 0.99 (95% CI: 0.97, 1.01). The relationship was shown by adopting the REM (tau-squared = 0.00, I^2^ = 96.8%, *p* < 0.001). Subgroup analyses of other factors were similar to the primary results and presented in the supplementary material (Supplemental Fig. S7-S9).

### Sensitivity analyses

Sensitivity analysis was conducted by excluding each study one by one to ensure the reliability of each study. Due to the high heterogeneity, REM was used. The results did not show significant change in these effect sizes, indicating the robustness of the results presented in our study (Supplemental Fig. S1-S3). We further removed the only study in T2D with OR as an outcome measure and the results remain robust (Supplemental Fig. S10).

### Publication bias

The Begg’s funnel-plot and Egger’s test were used to detect publication bias and the results are displayed in Supplemental Fig. S4-S6. No significant publication bias was detected in the T1D (Egger’s test, *p* = 0.910; Begg’s test, *p* = 1.000) and T2D (Egger’s test, *p* = 0.910; Begg’s test, *p* = 0.806) studies. The result from Egger’s test further suggested publication bias of GDM (*p* = 0.013), however, the Begg’s test indicated no statistical significance (*p* = 0.559). Potential causes of publication bias may include a tendency to report positive results, exaggerated publication bias due to difficult estimation of population heterogeneity, and a greater likelihood of publication bias in observational studies [37, 53]. This meta-analysis may overestimated the effect of ambient ozone exposure on diabetes due to publication bias.

## Discussion

Ozone is a common air pollutant, and its potential health hazard have gradually become a key public health concern. Our study analyzed existing evidence to evaluate the effects of ozone exposure on three types of diabetes including three studies on T1D risk involving 755,210 cases, five studies on T2D risk involving 8,322,170 cases, eleven studies on GDM risk involving 1,385,845 cases.We found that exposure to ozone (per 10 μg/m^3^ increase) was positively correlated with the risk of GDM, especially in pregnant women exposed to ozone during the first trimester of pregnancy. Additionally, ozone exposure was positively associated with risk for the development of T2D. However, no statistically significant association was found between ozone exposure and risk for the development of T1D.

In this meta-analysis, the exposure time to ozone in T1D was childhood and children’s age ranged from 0–18 years old. In T2D, the subjects were exposed to ozone for a long-term and their age distribution was widespread from 18 to over 75 years. In GDM, the research subjects’ exposure time was during or before pregnancy with 20–35 years of age. Most eligible studies were adjusted for multiple factors such as age, maternal age, race, education, birth year, and household income. Most of the included studies were conducted in America and China, others were in Canada and Italy.

Our results are consistent with most studies, but there are still some discrepancies. Elten et al. reported a negative correlation between ozone and T1D [11]. However, this study did not accurately distinguish between T1D and T2D suggesting that there was a possibility of bias, although the proportion of T2D in children is expected to be small. A cohort study by Li et al. did not observe an adverse effect of ozone on T2D [33]. Through comprehensive comparisons among  studies we extracted, we found that the absence of adjustments for socioeconomic status may be a major contributor to these discrepancies. A systematic review on the risk of ozone inhalation and adverse metabolic effects concluded that the current evidence is insufficient to conclude whether ozone exposure causes T1D and is insufficient or suggestive for the association with T2D [28]. As a result, more evidence is needed to explore these associations. Pan et al. surveyed the prevalence of GDM in the form of a questionnaire, which may reduce sensitivity and underestimate the role of ozone [41]. In a retrospective cohort study reported by Jo et al., ozone exposure was measured on a basis of the child's birth address rather than the residential geocoding of pregnant women, which may lead to information bias [23]. Despite the utilization of the REM model, significant heterogeneity was still found among studies during effect sizes combining. This may be attributed to the inconsistency in study design, exposure assessment, and adjustment of covariates. First, the number of studies on the effect of ozone exposure on T1D and T2D is limited. Moreover, most studies are retrospective studies, which may have introduced retrospective bias. Second, ozone concentrations vary greatly between indoor and outdoor environments [45]. However, in most studies included in our analysis, the data on ozone exposure was derived from outdoor fixed-site monitoring stations, which may not accurately reflect individual exposure levels [31, 46] especially for children with diabetes, the elderly or chronic patients, and pregnant women who may spend more time indoors. Models that relate indoor ozone concentrations to outdoor concentrations may be utilized to reduce this error [55]. Third, the various covariates in the literature included were unevenly distributed in different regions and populations, and the degree of control for potential confounding factors may be different, both can lead to bias. Although the statistics included in some studies were adjusted for similar factors, such as age, sex, ethnicity, smoking, etc., information errors still could not be completely ruled out.

The mechanism of ozone -induced diabetes was explored in both animal and molecular models. T2D is usually the result of β cell dysfunction in the context of chronic insulin resistance. Evidence has shown that ozone is a strong oxidant and produces reactive oxygen species (ROS), which can impact insulin-stimulated glucose uptake through oxidative stress response [15]. Oxidative stress has been confirmed as the basic mechanism for the pro-inflammatory response induced by air pollutants [57]. And the pro-inflammatory response is believed to promote the development of T2D [7]. Ozone activates transcription factors through ROS, mediating the NF-κB activation in ozone-exposed cells, which can increase the release of inflammatory cytokines (TNF-α and IL-8) and the expression of adhesion genes [6]. Bailey et al. suggested that exposure to ozone may induce changes in gut microbiota, which may contribute to the increased risk of T2D [3, 16]. GDM shares common pathogenic mechanisms with T2D [30], but in a special physiological state of pregnancy. Studies have shown that women may suffer a higher risk of T2D after GDM [61]. The placenta secretes hormones and cytokines, which contribute to the occurrence of reactions such as oxidative stress in the neuroendocrine system, resulting in insulin resistance [42]. In addition, Snow et al. showed that ozone exposure can excite the sympathetic nerve increasing the circulation of adrenal derived stress hormones [50], which leads to damage to the pancreas, fat, muscle tissue, and liver, ultimately contributing to the development of GDM through different mechanisms [42]. T1D is an autoimmune disease caused by insufficient insulin secretion and the destruction of pancreatic β cells [10]. Both genetic and non-genetic factors are likely to contribute to the development of T1D. The interaction between genetics and ozone exposure on initiation and development of T1D requires further exploration.

There are some strengths in this study. First, the size of the population sample contained in this study was relatively large. Second, our study covers countries at different levels of socio-economic development, and are thus a more representative sampling, avoiding unnecessary bias and improving the applicability of the results to most countries. Third, considering that the units of ozone are inconsistent, we referred to WHO to obtain standard unit conversion factors, in order to combine the effect values and we performed a logarithmic conversion to reduce heterogeneity. Fourth, since GDM is diagnosed in the middle and late trimester of pregnancy, the data obtained from the 1^st^ and 2^nd^ trimester of pregnancy account for a large proportion, which suggests the rationality in time sequence.

This study also has several limitations. First, in this study publication bias may exists. Selective reporting is unavoidable. It remains possible that studies measured more than one air pollutant including ozone in relation to diabetes risk, but only reported on positive associations potentially leaving out negative results on the association between ozone and diabetes risk, although some studies have reported negative results. Second, the number of articles included in this study was limited, however the size of the population sample contained was relatively large. Third, we only explored the effects of a single air pollutant on diabetes. However, some studies have shown that the single pollutant model is closer to reality than the composite pollutant model due to offsetting confounding and measurement errors [13].

## Conclusion

Ozone exposure was positively associated with T2D and GDM, especially during the first trimester of pregnancy, although the current studies were of low level on evidence grade. Therefore, more effective preventive measures and prenatal care to strengthen ozone exposure control are needed to improve the health of both adults and children. Future research is needed focused on the complex ozone-environment- diabetes interactions including the effects of mixed exposure reactions.

## Supplementary Information

**Additional file 1.**

## Data Availability

All data generated or analysed during this study are included in this published article [and its supplementary information files].
