# Supplementary material for: The effect of ambient ozone exposure on three types of diabetes: a meta-analysis
Source: Environ Health. 2023 Mar 30;22:32. doi: 10.1186/s12940-023-00981-0 (PMC10061724; doi:10.1186/s12940-023-00981-0)
Supplement: Supplementary file 1 — Additional file 1. [file 12940_2023_981_MOESM1_ESM.docx]

| Source | **Supplementary materials**  **Table S1** **Extracted effect values including source, exposure time, reported adjusted estimate (95%** **CI), outcome.**  Exposure  time | Reported fully adjusted estimate (95% CI) | Average ozone concentration (μg/m^3^) | O_3_ exposure assessment methods | GRADE | Outcome |
| --- | --- | --- | --- | --- | --- | --- |
| Elten et al. (2020) | childhood | HR= 0.89 (0.69, 1.14) per 3.31 ppb (IQR) | 50.78 | a spatiotemporal interpolation  technique | low | T1D |
| Hathout et al. (2002) | childhood | OR= 4.22 (1.96, 9.10) per 10.9 ppb | 63.60 | fixed-site monitoring stations in turn as receptor sites and interpolating from other stations | very low^a^ | T1D |
| Hathout et al. (2006) | childhood | OR= 1.73 (1.08, 2.77) per 10 ppb | 57.62 | fixed-site monitoring stations in turn as receptor sites and interpolating from other stations | very low^a^ | T1D |
| Jerrett et al. (2017) | - | HR= 1.18 (1.04, 1.34) per 6.7 ppb (IQR) | non | the Community Model for Air Quality (CMAQ) model | low | T2D |
| Li et al. (2021) | - | HR= 1.058 (1.053,1.064) per 3.3 ppb (IQR) | 51.37 | the ordinary kriging method implemented in ArcGIS Desktop  software (ESRI, Redland, CA, USA version 10.5) | low | T2D |
| Renzi et al. (2018) | - | HR= 1.015 (1.002, 1.027) per 10 μg/m^3^ | 97.40 | the Flexible Air quality Regional Model (FARM), a three-dimen- sional Eulerian model of the transport and multiphase chemistry of pollutants in the atmosphere | low | T2D |
| Yang et al. (2018) | - | OR= 1.14 (1.05, 1.25) per 22 μg/m^3^ | 49.40 | average | low | T2D |
| Yu et al. (2021) | - | HR= 1.13 (1.00, 1.28) per 10 ppb (IQR) | 90.16 | a land-use regression (LUR) model | low | T2D |
| Hu et al. (2015) | first trimester | OR= 1.09 (1.07, 1.11) per 5 ppb | 72.95 | the Community Multiscale Air Quality (CMAQ) model and the HBM approach | low | GDM |
|  | second trimester | OR= 1.12 (1.10, 1.14) per 5 ppb |  |  | low |  |
|  | entire pregnancy | OR= 1.18 (1.15, 1.21) per 5 ppb |  |  | low |  |
| Jo et al. (2019) | preconception | OR= 0.94 (0.92, 0.95) per 15,7 ppb | 80.95 | the distance-weighted approach | low | GDM |
|  | first trimester | OR= 0.95 (0.94, 0.97) per 15,7 ppb |  |  | low |  |
| Lin et al. (2020) | first trimester | OR= 1.01 (0.88, 1.15) per 10 μg/m^3^ | 55.30 | average | very low^b^ | GDM |
|  | second trimester | OR= 0.85 (0.75, 0.97) per 10 μg/m^3^ |  |  | low |  |
| Liu et al. (2022) | first trimester | OR= 1.05 (1.01, 1.10) per 10 μg/m^3^ | non | the inverse distance-weighted average | low | GDM |
|  | second trimester | OR= 1.02 (0.97, 1.07) per 10 μg/m^3^ |  |  | very low^b^ |  |
| Pan et al. (2017) | first trimester | OR= 0.97 (0.94, 0.99) per 1 ppb | 50.57 | ArcGIS Desktop v.10 (ESRI Inc., Redlands, CA, U.S.A.) with ordinary kriging | low | GDM |
|  | second trimester | OR= 0.97 (0.94, 1.00) per 1 ppb |  |  | low |  |
| Robledo et al. (2014) | preconception | OR= 0.93 (0.90, 0.96) per 12.33 ppb (IQR) | non | a modified Community Multi-scale Air Quality Model (CMAQ) | low | GDM |
|  | first trimester | OR= 1.00 (0.97, 1.03) per 12.36 ppb (IQR) |  |  | very low^b^ |  |
| Shen et al. (2017) | preconception | OR= 1.01 (0.96, 1.07) per 6.22 ppb (IQR) | 52.92 | the spatial analyst method (i.e., ordinary kriging) with the ArcGIS Desktop v.10 software | very low^b^ | GDM |
|  | first trimester | OR= 1.02 (0.96, 1.08) per 6.22 ppb (IQR) |  |  | very low^b^ |  |
|  | second trimester | OR= 1.04 (0.99, 1.11) per 6.22 ppb (IQR) |  |  | very low^b^ |  |
| Sun et al. (2022) | preconception | OR= 0.898 (0.886, 0.910) per 14.02 ppb (IQR) | 86.43 | empirical Bayesian kriging (EBK) | low | GDM |
|  | first trimester | OR= 0.938 (0.928, 0.947) per 13.94 ppb (IQR) |  |  | low |  |
|  | second trimester | OR= 0.909 (0.897, 0.921) per 13.74 ppb (IQR) |  |  | low |  |
|  | entire pregnancy | OR= 0.765 (0.748, 0.782) per 9.03 ppb (IQR) |  |  | low |  |
| Wu et al. (2016) | first trimester | OR= 1.019 (1.000, 1.038) per 9.96 ppb (IQR) | 82.52 | empirical Bayesian kriging (EBK) | low | GDM |
|  | second trimester | OR= 1.004 (0.983, 1.026) per 9.96 ppb (IQR) |  |  | very low^b^ |  |
| Yao et al. (2020) | preconception | OR= 1.19 (1.08, 1.31) per 50.29 μg/m^3^ (IQR) | non | average | low | GDM |
|  | first trimester | OR= 1.26 (1.13, 1.42) per 46.39 μg/m^3^ (IQR) |  |  | low |  |
| Yan et al. (2022) | first trimester | OR= 1.013 (0.945, 1.086) per 10 μg/m^3^ | 93.8 | the atmospheric chemical transport model (CTM) | very low^b^ | GDM |
|  | second trimester | OR= 1.045 (0.992, 1.100) per 10 μg/m^3^ |  |  | very low^b^ |  |
|  | entire pregnancy | OR= 0.987 (0.904, 1.077) per 10 μg/m^3^ |  |  | very low^b^ |  |

1. Downgraded one level for imprecision. The sample size is small。
2. Downgraded one level for imprecision. At opposite ends of the 95% confidence interval, different conclusions are drawn.

**Table S2 PRISMA checklist**

| **Section and Topic** | **Item #** | **Checklist item** | **Location where item is reported** |
| --- | --- | --- | --- |
| **TITLE** | | |  |
| Title | 1 | Identify the report as a systematic review. | Title |
| **ABSTRACT** | | |  |
| Abstract | 2 | See the PRISMA 2020 for Abstracts checklist. | Abstract |
| **INTRODUCTION** | | |  |
| Rationale | 3 | Describe the rationale for the review in the context of existing knowledge. | 1. Introduction (line 64-104 ) |
| Objectives | 4 | Provide an explicit statement of the objective(s) or question(s) the review addresses. | 1. Introduction (line 105-108) |
| **METHODS** | | |  |
| Eligibility criteria | 5 | Specify the inclusion and exclusion criteria for the review and how studies were grouped for the syntheses. | 2.2. Selection criteria (line 129-145) |
| Information sources | 6 | Specify all databases, registers, websites, organisations, reference lists and other sources searched or consulted to identify studies. Specify the date when each source was last searched or consulted. | 2.1. Search methods (line 112-113, 127-128) |
| Search strategy | 7 | Present the full search strategies for all databases, registers and websites, including any filters and limits used. | 2.1. Search methods (line 119-126) |
| Selection process | 8 | Specify the methods used to decide whether a study met the inclusion criteria of the review, including how many reviewers screened each record and each report retrieved, whether they worked independently, and if applicable, details of automation tools used in the process. | 2.3. Study screening and data extraction (line 146-150) |
| Data collection process | 9 | Specify the methods used to collect data from reports, including how many reviewers collected data from each report, whether they worked independently, any processes for obtaining or confirming data from study investigators, and if applicable, details of automation tools used in the process. | 2.3. Study screening and data extraction (line 146-150) |
| Data items | 10a | List and define all outcomes for which data were sought. Specify whether all results that were compatible with each outcome domain in each study were sought (e.g. for all measures, time points, analyses), and if not, the methods used to decide which results to collect. | 2.3. Study screening and data extraction (line 150-152) |
|  | 10b | List and define all other variables for which data were sought (e.g. participant and intervention characteristics, funding sources). Describe any assumptions made about any missing or unclear information. | 2.3. Study screening and data extraction (line 150-152) |
| Study risk of bias assessment | 11 | Specify the methods used to assess risk of bias in the included studies, including details of the tool(s) used, how many reviewers assessed each study and whether they worked independently, and if applicable, details of automation tools used in the process. | 2.4. Quality assessment (line 154-159) |
| Effect measures | 12 | Specify for each outcome the effect measure(s) (e.g. risk ratio, mean difference) used in the synthesis or presentation of results. | 2.2. Selection criteria (line 137-138) |
| Synthesis methods | 13a | Describe the processes used to decide which studies were eligible for each synthesis (e.g. tabulating the study intervention characteristics and comparing against the planned groups for each synthesis (item #5)). | Table 1 |
|  | 13b | Describe any methods required to prepare the data for presentation or synthesis, such as handling of missing summary statistics, or data conversions. | 2.5. Statistical methods (line 161-171) |
|  | 13c | Describe any methods used to tabulate or visually display results of individual studies and syntheses. | 2.5. Statistical methods (line 161-171) |
|  | 13d | Describe any methods used to synthesize results and provide a rationale for the choice(s). If meta-analysis was performed, describe the model(s), method(s) to identify the presence and extent of statistical heterogeneity, and software package(s) used. | 2.5. Statistical methods (line 161,172 -177) |
|  | 13e | Describe any methods used to explore possible causes of heterogeneity among study results (e.g. subgroup analysis, meta-regression). | 2.5. Statistical methods (line 177-179) |
|  | 13f | Describe any sensitivity analyses conducted to assess robustness of the synthesized results. | 2.5. Statistical methods (line 181-183) |
| Reporting bias assessment | 14 | Describe any methods used to assess risk of bias due to missing results in a synthesis (arising from reporting biases). | 2.5. Statistical methods (line 179-181) |
| Certainty assessment | 15 | Describe any methods used to assess certainty (or confidence) in the body of evidence for an outcome. | GRADE |
| **RESULTS** | | |  |
| Study selection | 16a | Describe the results of the search and selection process, from the number of records identified in the search to the number of studies included in the review, ideally using a flow diagram. | 3.1. Study search results (line 186-210) |
|  | 16b | Cite studies that might appear to meet the inclusion criteria, but which were excluded, and explain why they were excluded. | 3.1. Study search results (line 186-210) |
| Study characteristics | 17 | Cite each included study and present its characteristics. | 3.2. Characteristics overview (line 211- 237), Table 1 |
| Risk of bias in studies | 18 | Present assessments of risk of bias for each included study. | 3.5. Publication bias (line 284-289) |
| Results of individual studies | 19 | For all outcomes, present, for each study: (a) summary statistics for each group (where appropriate) and (b) an effect estimate and its precision (e.g. confidence/credible interval), ideally using structured tables or plots. | Table S1 |
| Results of syntheses | 20a | For each synthesis, briefly summarise the characteristics and risk of bias among contributing studies. | 3.3. Meta- analysis on ozone exposure and the risk of diabetes (line 240-273) |
|  | 20b | Present results of all statistical syntheses conducted. If meta-analysis was done, present for each the summary estimate and its precision (e.g. confidence/credible interval) and measures of statistical heterogeneity. If comparing groups, describe the direction of the effect. | 3.3. Meta- analysis on ozone exposure and the risk of diabetes (line 240-273) |
|  | 20c | Present results of all investigations of possible causes of heterogeneity among study results. | 3.3. Meta- analysis on ozone exposure and the risk of diabetes (line 240-273) |
|  | 20d | Present results of all sensitivity analyses conducted to assess the robustness of the synthesized results. | 3.4. Sensitivity analyses (line 275-278) |
| Reporting biases | 21 | Present assessments of risk of bias due to missing results (arising from reporting biases) for each synthesis assessed. | 3.5. Publication bias (line 285-290) |
| Certainty of evidence | 22 | Present assessments of certainty (or confidence) in the body of evidence for each outcome assessed. |  |
| **DISCUSSION** | | |  |
| Discussion | 23a | Provide a general interpretation of the results in the context of other evidence. | Discussion (line 293-328) |
|  | 23b | Discuss any limitations of the evidence included in the review. | Discussion (line 328-345, 383-393) |
|  | 23c | Discuss any limitations of the review processes used. | Discussion (line 312-328) |
|  | 23d | Discuss implications of the results for practice, policy, and future research. | Discussion (line 346-382), conclusion |
| **OTHER INFORMATION** | | |  |
| Registration and protocol | 24a | Provide registration information for the review, including register name and registration number, or state that the review was not registered. | Not registered |
|  | 24b | Indicate where the review protocol can be accessed, or state that a protocol was not prepared. | Not registered |
|  | 24c | Describe and explain any amendments to information provided at registration or in the protocol. | Not registered |
| Support | 25 | Describe sources of financial or non-financial support for the review, and the role of the funders or sponsors in the review. | Funding |
| Competing interests | 26 | Declare any competing interests of review authors. | Competing interests |
| Availability of data, code and other materials | 27 | Report which of the following are publicly available and where they can be found: template data collection forms; data extracted from included studies; data used for all analyses; analytic code; any other materials used in the review. | Included studies: Table 1  Data used for all analyses: Table S1  Analytic code: Willing to disclose the data, please contact the corresponding author if there is a further need |


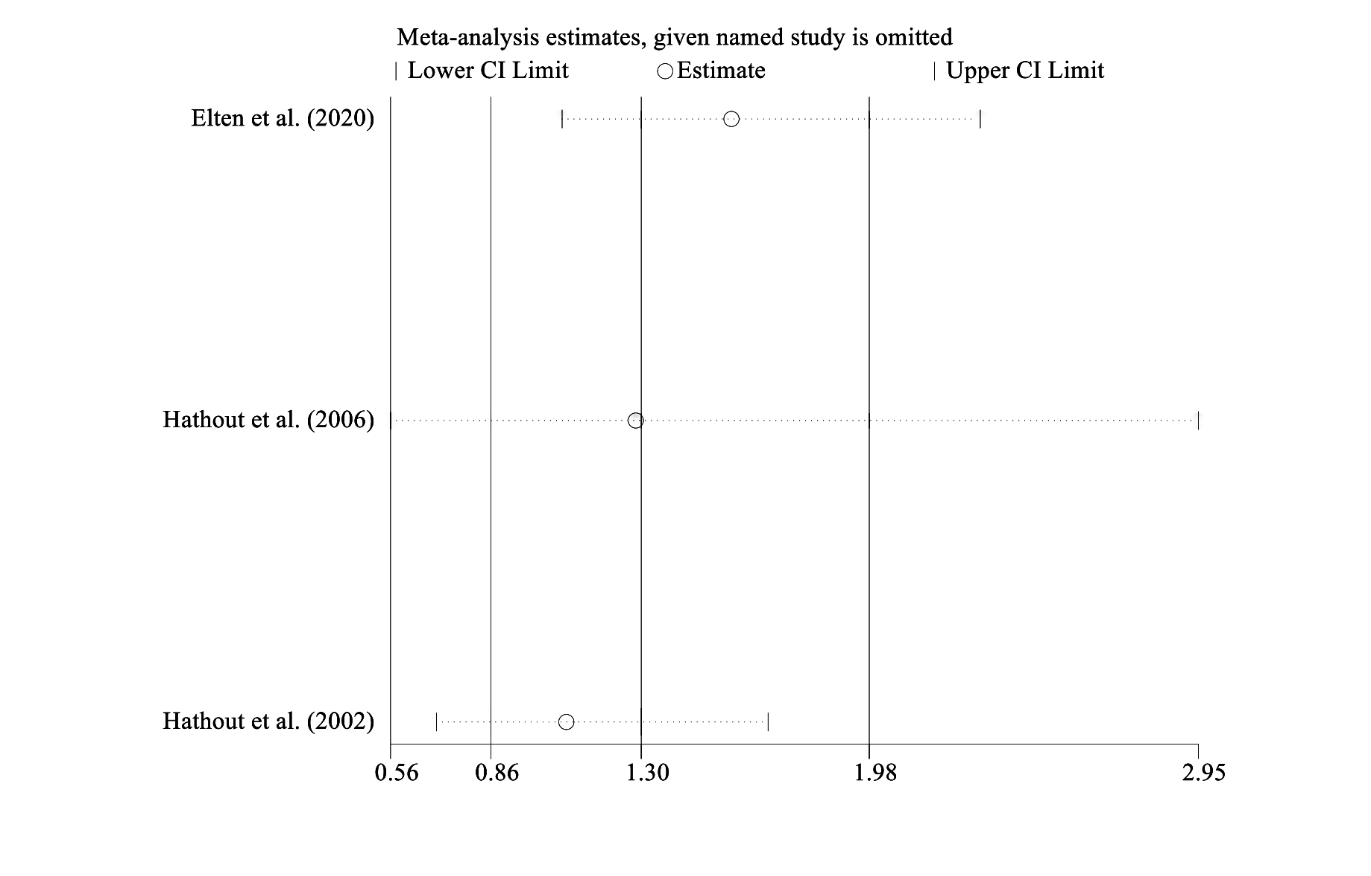


**Fig S1. Sensitivity analysis for T1D**


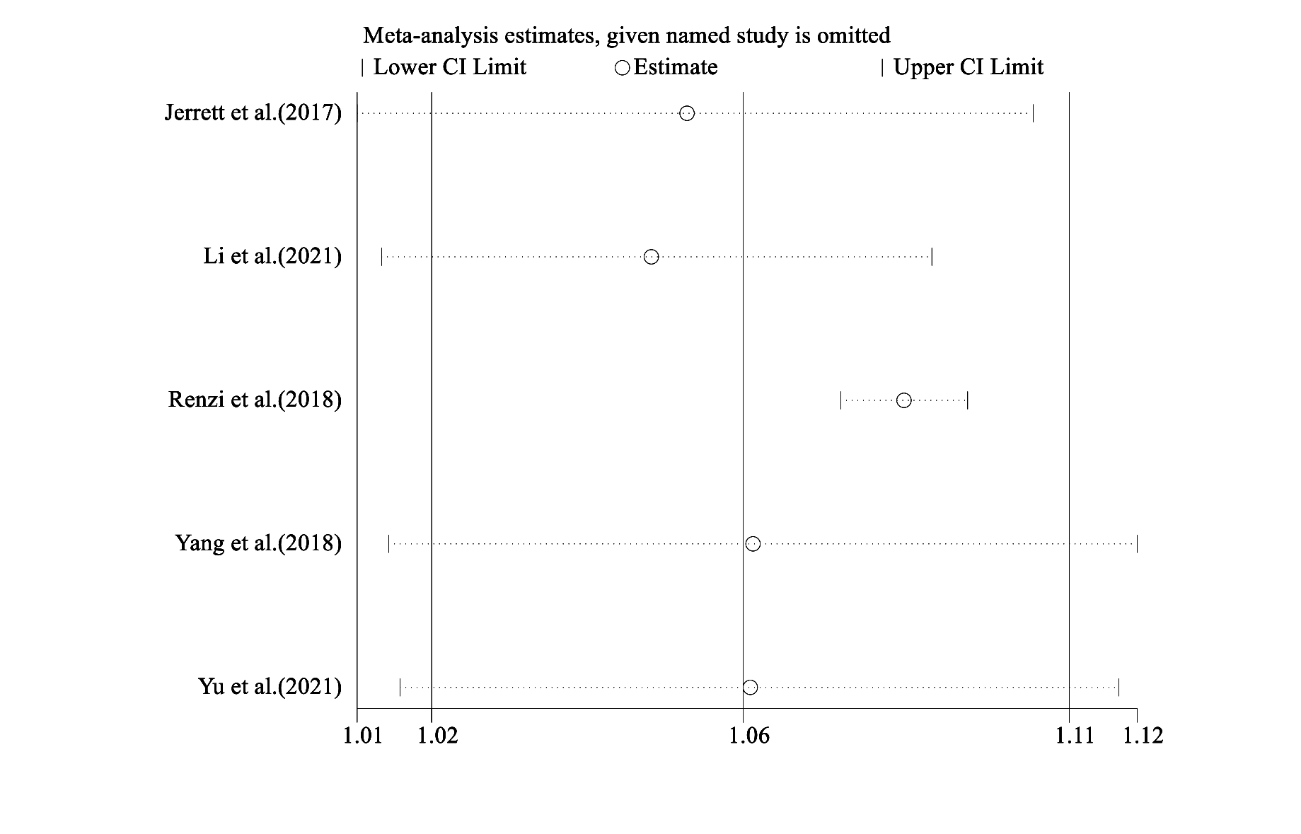


**Fig S2. Sensitivity analysis for T2D**


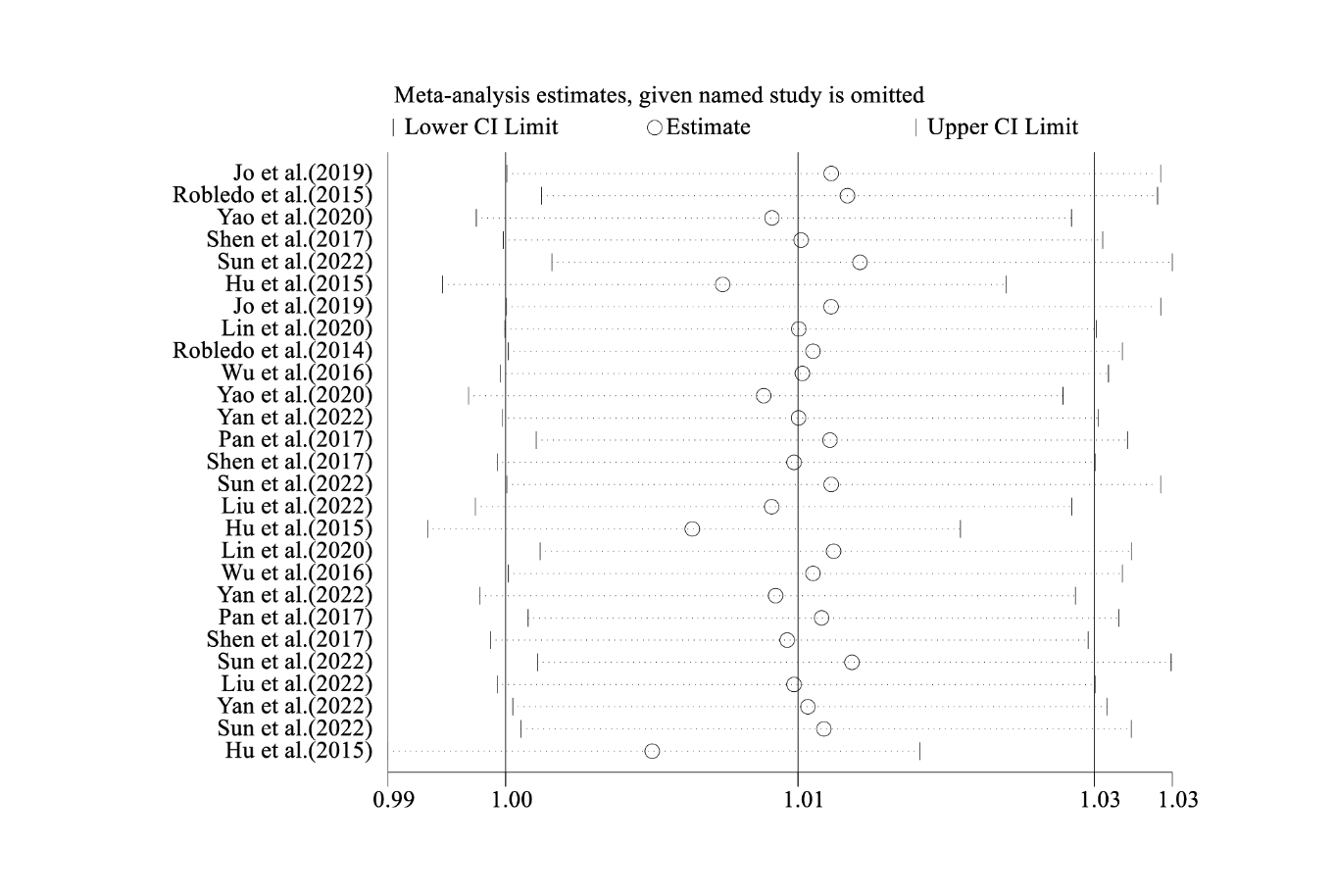


**Fig S3. Sensitivity analysis for GDM**


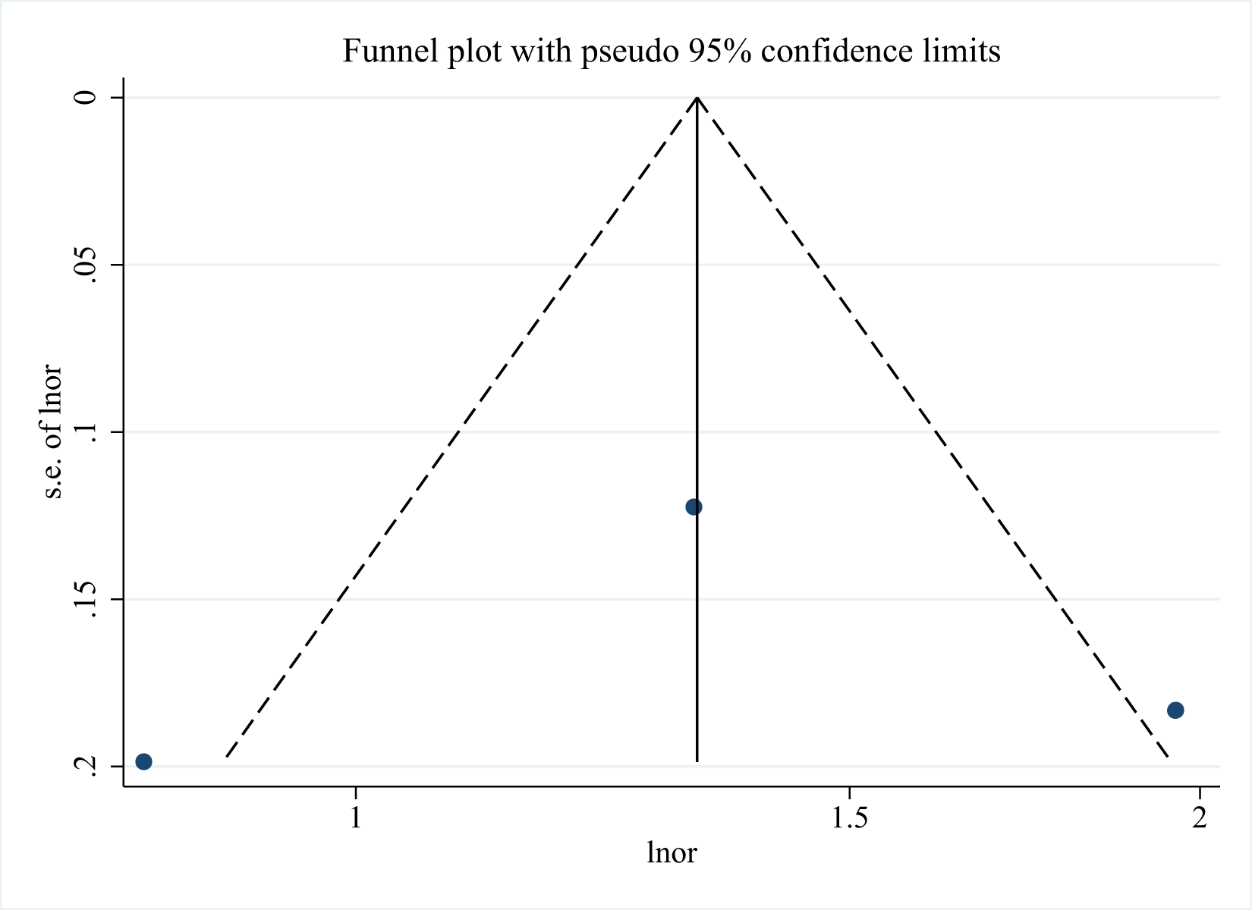


**Fig S4. Funnel chart for publication bias.** **There is no apparent publication bias for T1D (p > 0.05)**


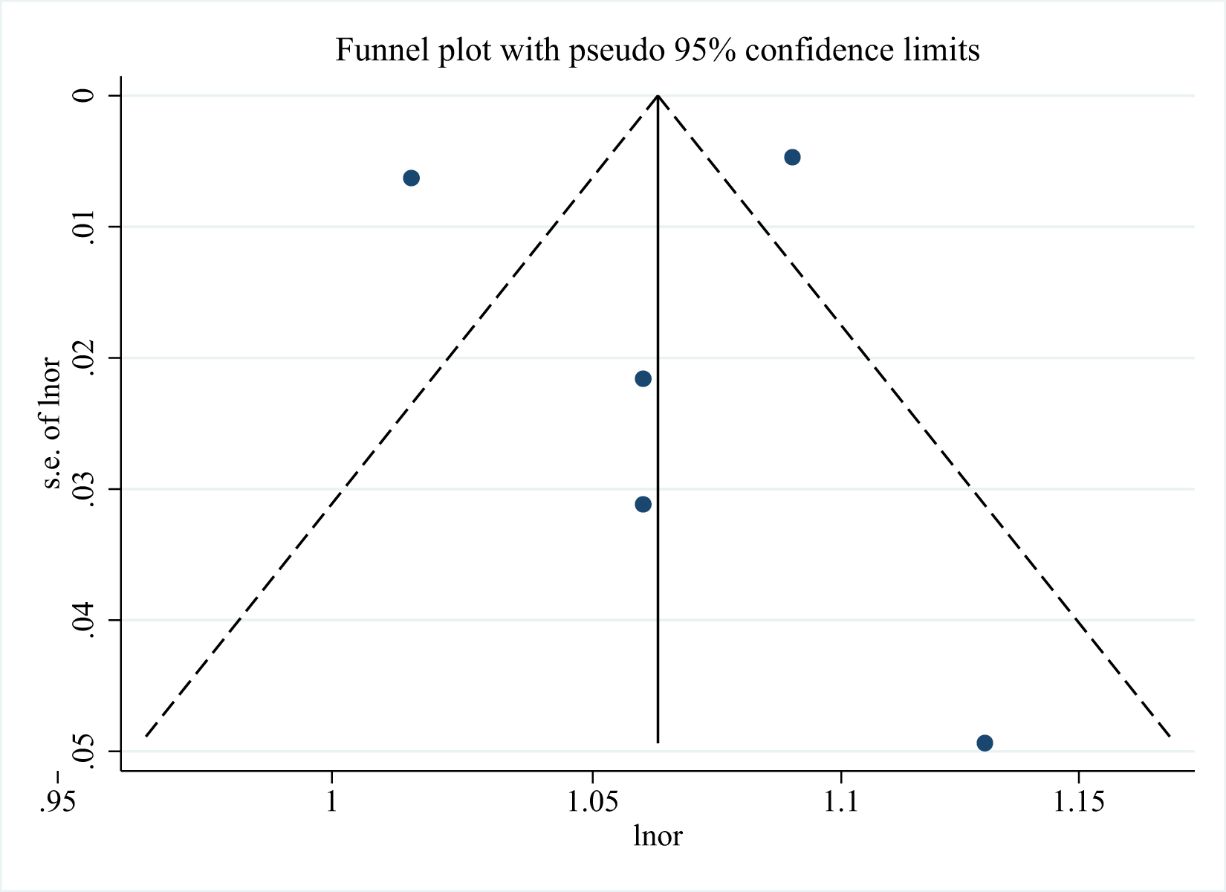


**Fig S5.** **Funnel chart for publication bias. There is no apparent publication bias for T2D (p > 0.05)**


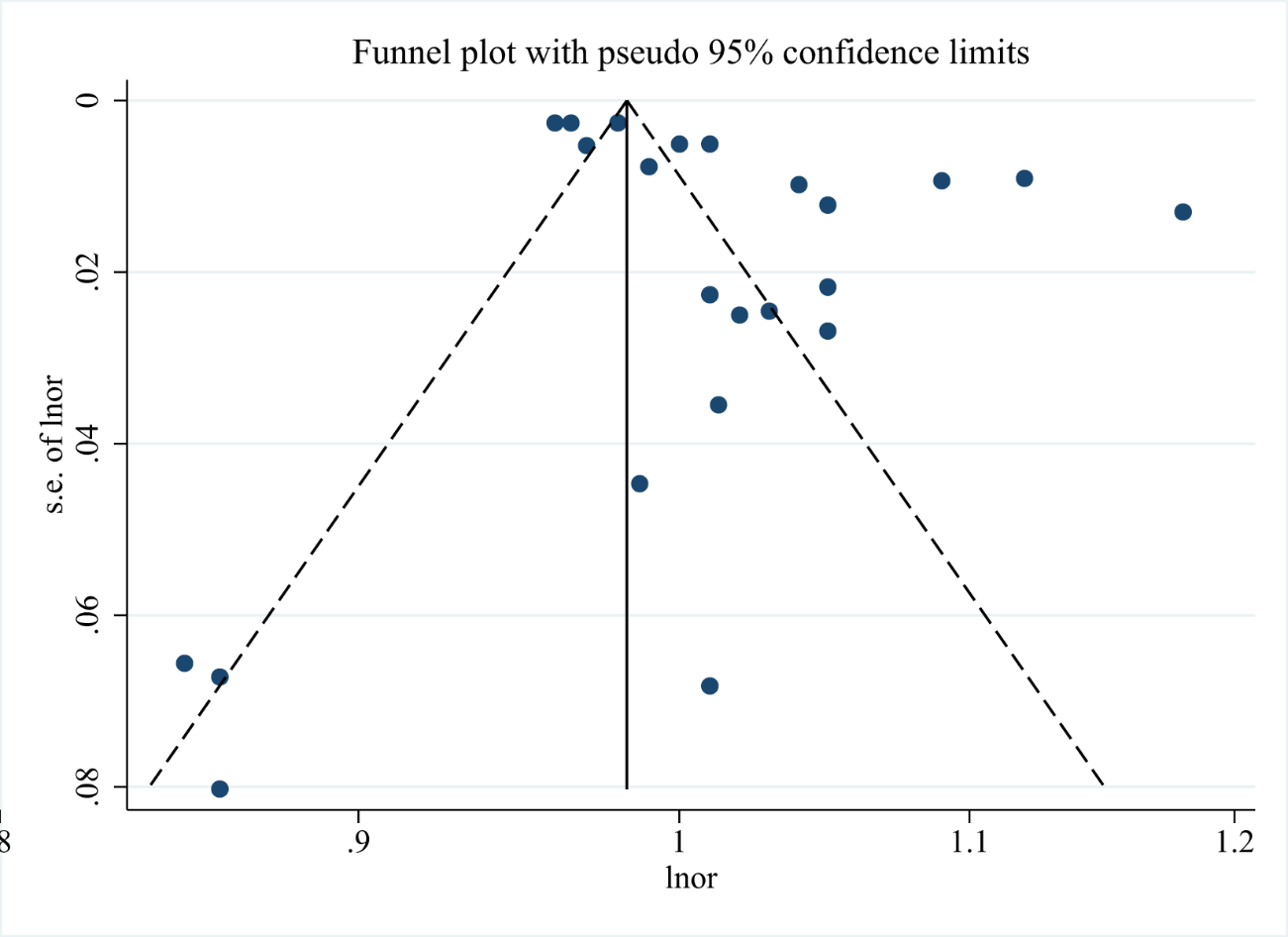


**Fig S6.** **Funnel chart for publication bias. There is a possibility of publication bias for GDM (p < 0.05).**

**Fig S7. The relationship between ozone exposure in early pregnancy and GDM was analyzed in a subgroup , with the ozone exposure measurement method and the model used as factors.**

Note: We extracted ozone exposure assessment methods from the original text of each study, with the main assessment methods including using the atmospheric chemical transport model (CTM), taking the average directly, spatial interpolation.

1. The atmospheric chemical transport model (CTM): the Community Multiscale Air Quality (CMAQ) model.
2. Spatial interpolation: the empirical Bayesian kriging (EBK), the distance-weighted approach or ArcGIS Desktop v.10 (ESRI Inc., Redlands, CA, U.S.A.).
3. For different evaluation methods, we performed subgroup analyses, and the results showed significant association with OR = 1.02 (95% CI: 1.00, 1.03) between the first trimester ozone exposure and GDM. Moreover, three articles that used CTM, six articles that performed spatial interpolation, were marginal significance with OR = 1.04 (95% CI: 0.97, 1.11) and OR = 0.99 (95% CI: 0.98, 1.01) respectively. The effect sizes of the two articles taking average revealed significant association with OR = 1.05 (95% CI: 1.02, 1.07). The relationship was shown by adopting the REM (I^2^ = 94.8%, p < 0.001).

**A**

**B**

**Fig S8. The relationship between ozone exposure and T2D, GDM was analyzed in a subgroup , with the average concentration of ozone exposure as factor.**

A. T2D and ozone exposure

B. GDM and ozone exposure during the first trimester

Note: a. Ozone levels: According to the AQG 2021 published by WHO, a peak seasonal average of O_3_ concentrations was added to 60 μg/m^3^ based on evidence of health effects linking long-term ozone concentrations to total mortality and respiratory mortality. We divided subgroups according to the ozone exposure mean value in T2D, with those below 60 μg/m^3^ listed as low levels and those above 60 μg/m^3^as high levels. However, one study did not give an average level of ozone exposure.

b.In T2D, the study in areas with low average ozone exposure found significant positive effects with OR = 1.08 (95% CI: 1.06, 1.11), but no significant effect in areas with higher levels with OR = 1.03 (95% CI: 0.99, 1.06). In GDM, there was no significant change in heterogeneity.

**A**

**B**

**Fig S9. Based on the prevailing criteria, we categorized the countries and regions covered in the included studies as developed and developing, and performed a subgroup analysis of the data in T2D and the first trimester in GDM.**

A. T2D and ozone exposure

B. GDM and ozone exposure during the first trimester

Note: In T2D, there was no significant change in heterogeneity. In GDM, the study in development areas found significant positive effects with OR = 1.01 (95% CI: 0.99, 1.03), but no significant effect in developed areas with OR = 1.05 (95% CI: 1.03, 1.07).

**
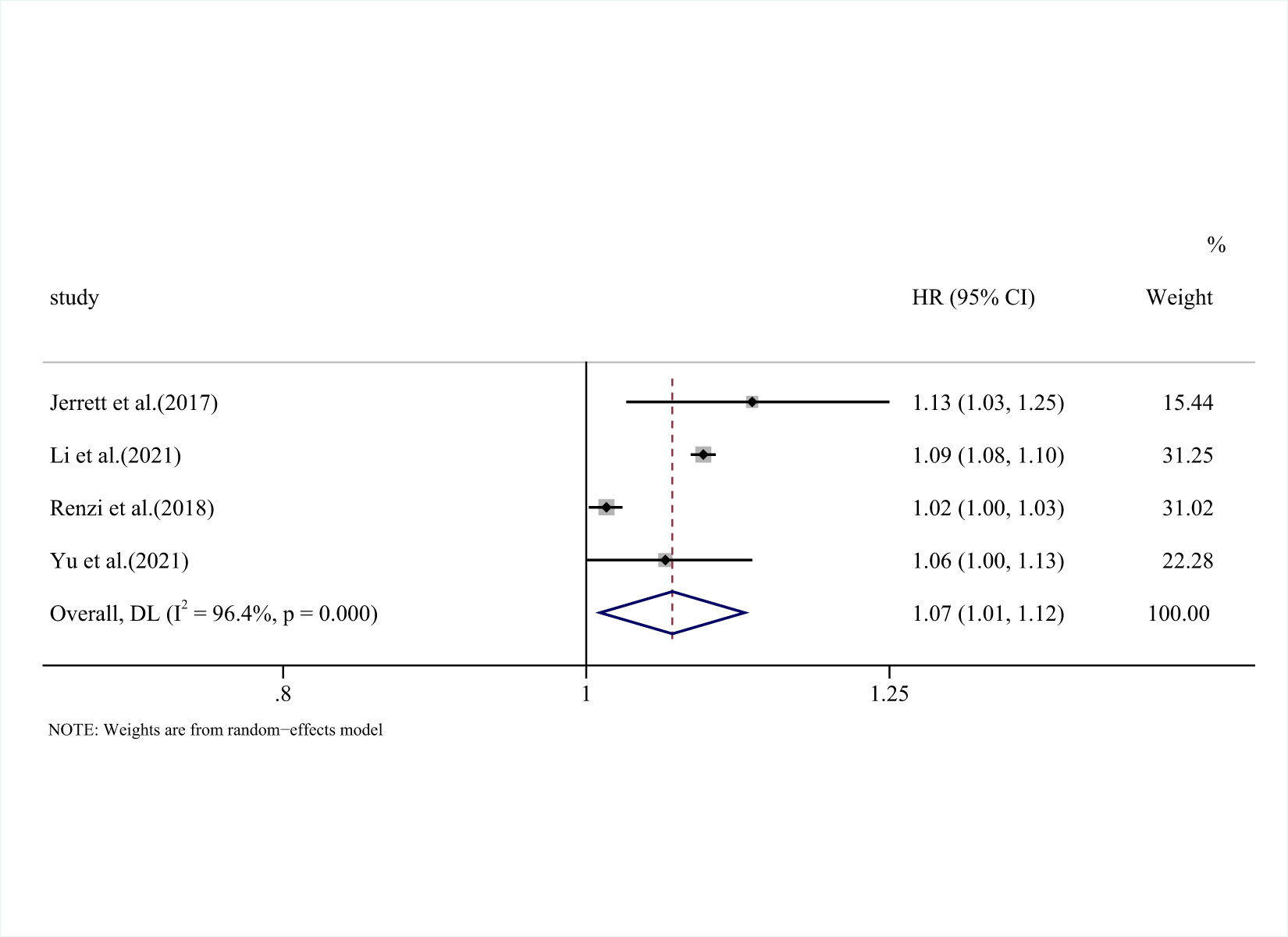
**

**Fig S10. Sensitivity analyses: literature with different effect measures was removed in T2D.**
